# Nicotinic acetylcholine receptors induce c-Kit ligand/Stem Cell Factor and promote stemness in an ARRB1/ β-arrestin-1 dependent manner in NSCLC

**DOI:** 10.18632/oncotarget.2395

**Published:** 2014-08-27

**Authors:** Deepak Perumal, Smitha Pillai, Jonathan Nguyen, Courtney Schaal, Domenico Coppola, Srikumar P. Chellappan

**Affiliations:** ^1^ Department of Tumor Biology, H. Lee Moffitt Cancer Center & Research Institute, Magnolia Drive, Tampa, FL, USA; ^2^ Department of Anatomic Pathology, H. Lee Moffitt Cancer Center & Research Institute, Magnolia Drive, Tampa, FL, USA; ^3^ Department of Hematology and Medical Oncology, Icahn School of Medicine at Mount Sinai, New York, USA

**Keywords:** Self-renewal, E2F1, non-small cell lung cancer, Rb

## Abstract

Lung cancer remains the leading cause of cancer-related deaths worldwide. β-arrestin-1 (ARRB1), a scaffolding protein involved in the desensitization of signals arising from activated G-protein-coupled receptors (GPCRs), has been shown to play a role in invasion and proliferation of cancer cells, including nicotine-induced proliferation of human non–small cell lung cancers (NSCLCs). In this study, we identified genes that are differentially regulated by nicotine in an ARRB1/β-arrestin-1 dependent manner in NSCLC cells by microarray analysis. Among the identified genes, SCF (Stem cell factor) strongly differentiated smokers from non-smokers in the Director's Challenge Set expression data and its high expression correlated with poor prognosis. SCF, a major cytokine is the ligand for the c-Kit proto-oncogene and was found to be over expressed in human lung adenocarcinomas, but not squamous cell carcinomas. Data presented here show that transcription factor E2F1 can induce SCF expression at the transcriptional level and depletion of E2F1 or ARRB1/β-arrestin-1 could not promote self-renewal of SP cells. These studies suggest that nicotine might be promoting NSCLC growth and metastasis by inducing the secretion of SCF, and raise the possibility that targeting signalling cascades that activate E2F1 might be an effective way to combat NSCLC.

## INTRODUCTION

Lung cancer is the leading cause of cancer-related mortality accounting for approximately 15% of all cancer cases worldwide and has a 5-year survival rate of 15% [1]. Non-Small Cell Lung Cancer (NSCLC) accounts for approximately 85% of all lung cancer cases and is highly correlated with tobacco smoking [2]. Nicotine is the major addictive component of cigarette smoke and acts by binding to nicotinic acetylcholine receptors (nAChRs) [3] that are expressed on neurons and neuromuscular junctions. In addition to promoting addiction to tobacco, exposure of non-neuronal cells to nicotine has been shown to promote cell proliferation and angiogenesis, enhancing tumor growth and metastasis [4-6]. nAChRs are among the most well understood receptors [7] and have emerged as key therapeutic targets for a variety of disorders [8-10]. Studies have shown that binding of nicotine to nAChRs triggers the synthesis and release of the stress neurotransmitters norepinephrine and epinephrine from lung adenocarcinoma cells and small airway epithelial cells. This stress-neurotransmitter-induced β-adrenergic receptor-initiates signaling via multiple pathways, including ERK, EGFR, Src and AKT resulting in proliferation and migration of these cells [11]. It has been reported that xenografts from lung adenocarcinomas are promoted in their growth via these β-adrenergic receptor pathways when the mice were exposed to social stress, resulting in elevated systemic levels of stress neurotransmitters [12].

Our earlier studies have shown that the binding of nicotine to nAChRs leads to the formation of an oligomeric complex between nAChR, Src, and β-arrestin-1 (ARRB1), promoting the prolif­eration of NSCLC cells [13]. ARRB1/β-arrestin-1 is a multifunctional scaffolding protein involved in the desensitization of activated G-protein-coupled receptors [14]. Proliferative sig­naling via the α7-nAChRs induced the phosphorylation of the retinoblastoma tumor suppressor protein, Rb, leading to its dissociation from E2F transcrip­tion factor 1 (E2F1)[15], promoting cell cycle progression. Not surprisingly, the Rb-E2F pathway is deregulated in about 90% of lung cancers [16]. Recent study has shown improved survival outcomes with the incidental use of beta-blockers among patients with NSCLC, including adenocarcinoma [17]. Another study has reported that blockade of the β2AR-β-arrestin-1 signaling cascade prevents the accumulation of DNA damage caused in mice by chronic restraint stress [18]. Given this background, attempts were made to identify nicotine induced, β-arrestin-1 dependent genes to assess their role in lung cancer. Towards this purpose, a microarray analysis was conducted on cells lacking ARRB1/β-arrestin-1, which have been rendered quiescent and subsequently stimulated with nicotine. One of the identified genes, *SCF* (Stem cell factor/c-Kit ligand), strongly differentiated smokers from non-smokers, suggesting a role of this gene in lung carcinogenesis induced by smoking. SCF is known to promote the self-renewal, proliferation and differentiation of numerous embryonic,[19, 20] adult hematopoietic,[21] neural[22] and primordial[23] stem cells, together with its receptor c-Kit [24].

An examination of the molecular mechanisms underlying the expression of SCF in NSCLC cell lines showed that the *SCF* promoter has multiple E2F binding sites and is induced by nicotine and EGF in a ARRB1/β-arrestin-1 dependent manner. Further, conditioned media from nicotine stimulated cells promoted the self-renewal of stem-like side population (SP) cells from NSCLC in a sphere-formation assay; interestingly, conditioned media from cells lacking β-arrestin-1 or E2F1 was unable to promote self-renewal. These results raise the possibility that exposure to nicotine or similar tobacco components might promote the growth of NSCLC by regulating the self-renewal and differentiation of stem-like cells.

## RESULTS

### Microarray analysis and prognosis prediction

**Figure 1:**
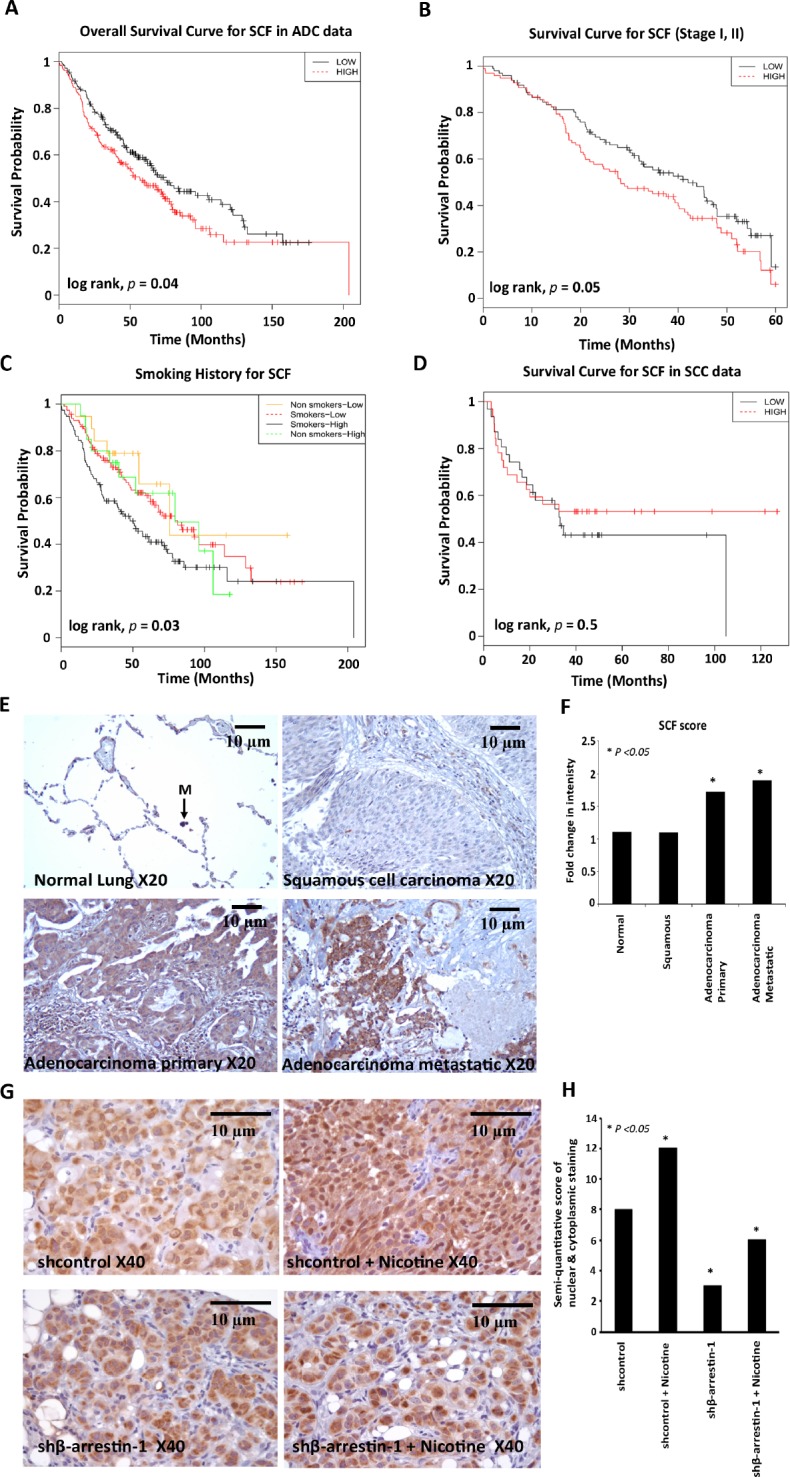
High levels of SCF correlates with poor survival in lung adenocarcinoma (A) Overall survival curve for SCF in 360 lung adenocarcinoma samples from the NCI Directors Challenge Set (B) Kaplan-Meier survival curve for SCF in stage I, II patients from the NCI Directors Challenge Set, (C) Overall survival curve for SCF according to smoking history in the NCI Directors Challenge Set (p value is significant between smokers with low and high SCF expression) (D) Recurrence free survival curve for SCF in squamous cell carcinoma data (n = 75) from the SKKU (Sungkyunkwan University) dataset. (E) IHC staining of SCF in Human Lung Cancer TMA using anti-human SCF antibody; representative images of SCF expression in normal lung tissue (M, Macrophage), squamous cell carcinomas, adenocarcinomas and metastatic carcinomas are shown. Magnification 20X, scale bar = 10μm. (F) Quantification of SCF immunostaining. (G) IHC staining of SCF from mice lung tumor sections revealed that SCF expression was significantly higher in tumors from nicotine treated mice (shcontrol nicotine) compared to tumors from vehicle treated mice implanted with shcontrol cells. SCF expression was significantly reduced in the β-arrestin-1 depleted cells (shβ-arrestin-1 and shβ-arrestin-1 nicotine) as compared to Shcontrol cells. Magnification 40X, scale bar = 10μm (H) Quantification of SCF immunostaining.

A549 cells transfected with a control non-targeting siRNA or a siRNA targeting β-arrestin-1 were rendered quiescent and subsequently stimulated with nicotine. A microarray analysis was performed and the mRNA expression profiles were measured using Affymetrix Expression Console™ software. We identified 296 genes that were upregulated and 208 that were down regulated by nicotine in an ARRB1/β-arrestin-1 dependent fashion. We selected the top 10 genes that were up- and down- regulated and assessed whether their expression could predict prognosis of NSCLC patients (Table 1A and B). Prognostic prediction was carried out on a subset of NCI Director's Challenge Set [25]. Kaplan-Meier analyses for 5 year as well as overall survival showed significance for 4 genes namely *COL4A4*, *NFASC*, *SCF* and *ZNF137* by log-rank test. We also examined whether the expression of these genes correlated with smoking; it was found that only *SCF* strongly differentiated smokers from non-smokers implying a potentially important role for this gene in lung carcinogenesis induced by smoking. Although *COL4A4*, *NFASC* and *ZNF137* show significant prognosis for overall survival and stage I, II in lung adenocarcinoma they failed to predict prognosis while correlating with the smoking history. Prognosis for *SCF* shown here is specific for adenocarcinomas, since a similar analysis conducted on 75 squamous cell carcinoma profiles from the SKKU dataset [26] showed no significant correlation with survival (Figure 1A-D). This suggests a specific role for SCF in the biology of lung adenocarcinomas.

**Table 1:** Microarray was performed in ARRB1 depleted and nicotine stimulated A549 cells Nicotine induced and ARRB1 dependent genes from the microarray data were analyzed. We identified differentially regulated genes that were regulated by nicotine in a β-arrestin-1 dependent fashion and top 10 up/down regulated genes from the list were used for prognosis prediction. Assessment of the expression of these genes for smoking revealed that SCF (highlighted in red) strongly differentiated smokers from non-smokers implying an important role of this gene in lung carcinogenesis induced by smoking

1A	Nicotine induced β-Arrestin-1 (ARRB1) dependent genes				
S.No	Probe ID	GenBank ID	Gene Symbol	Control si Nic vs Control si SS	ARRB1 si Nic vs Control si SS
1	1566324_a_at	NM_001031804.1	MAF	27.86	14.86
2	214524_at	NM_021081.3	GHRH	16.22	3.30
3	214793_at	X93921	DUSP7	12.29	1.90
4	214602_at	NM_000092.3	COL4A4	13.92	3.58
5	213438_at	NM_015090.2	NFASC	13.36	4.07
6	222176_at	AK021487	PTEN	11.00	2.08
7	220337_at	NM_021257.3	NGB	10.66	1.77
8	208542_x_at	NM_007153.1	ZNF208	9.83	1.39
9	210289_at	NM_003960.2	NAT8	9.48	1.30
10	211124_s_at	NM_000899.3	SCF	8.88	1.38

1B	Nicotine down regulated β-Arrestin-1 (ARRB1) dependent genes				
S.No	Probe ID	GenBank ID	Gene Symbol	Control si Nic vs Control si SS	ARRB1 si Nic vs Control si SS
1	206626_x_at	NM_005635.2	SSX1	−17.74	−1.01
2	219943_s_at	NM_022741	FLJ11850	−17.68	−1.48
3	206733_at	NM_003323.1	TULP2	−17.10	−2.58
4	207394_at	NM_003438.2	ZNF137	−15.33	−1.43
5	211887_x_at	NM_002445	MSR1	−15.21	−1.49
6	205848_at	NM_005256.2	GAS2	−14.58	−1.21
7	217507_at	NM_000578	SLC11A1	−13.91	−2.06
8	207236_at	NM_003419.2	ZNF345	−13.36	−6.45
9	207523_at	NM_006781.2	C6orf10	−13.10	−1.49
10	211227_s_at	NM_032971.1	PCDH11Y	−11.85	−1.50

### SCF levels are elevated in adenocarcinomas, but not squamous cell carcinomas of the lung

Since *SCF* message levels correlated with poor prognosis, we examined whether levels of SCF is altered in human lung cancer. Towards this purpose, human lung cancer tissue microarrays were immunostained using a rabbit anti-human SCF antibody. It was found that SCF levels were elevated in primary lung adenocarcinoma and metastatic carcinomas compared to normal lung tissues (Figure 1E); SCF levels were not elevated in primary squamous cell carcinomas (Figure 1F). Taken together, these results indicate that ele­vated levels of SCF may contribute at least, in part, to the growth and metastasis of lung adenocarcinomas.

In addition to strengthen SCF dependence on ARRB1/β-arrrestin-1 and nicotine, we performed IHC for SCF from mice lung tumor sections implanted with β-arrestin-1 depleted cells (shβ-arrestin-1). The lung tumor sections were prepared from a previously performed experiment (data to be published) in which shcontrol A549 cells or shβ-arrestin-1 cells were implanted orthotopically into athymic nude mice and the mice were administered PBS or nicotine for 6 weeks to observe growth of tumors. IHC staining of SCF with these sections (Figure 1G and H) revealed that SCF expression was significantly higher in tumors from nicotine treated mice (shcontrol nicotine) compared to tumors from vehicle treated mice implanted with shcontrol cells. SCF expression was significantly reduced in the ARRB1/β-arrestin-1 depleted cells (shβ-arrestin-1 and shβ-arrestin-1 nicotine ) as compared to shcontrol cells. The IHC results confirm again that ARRB1/β-arrestin-1 is necessary for SCF induction by nicotine.

### Nicotine and EGF induce SCF expression in a β-Arrestin-1 dependent manner

**Figure 2:**
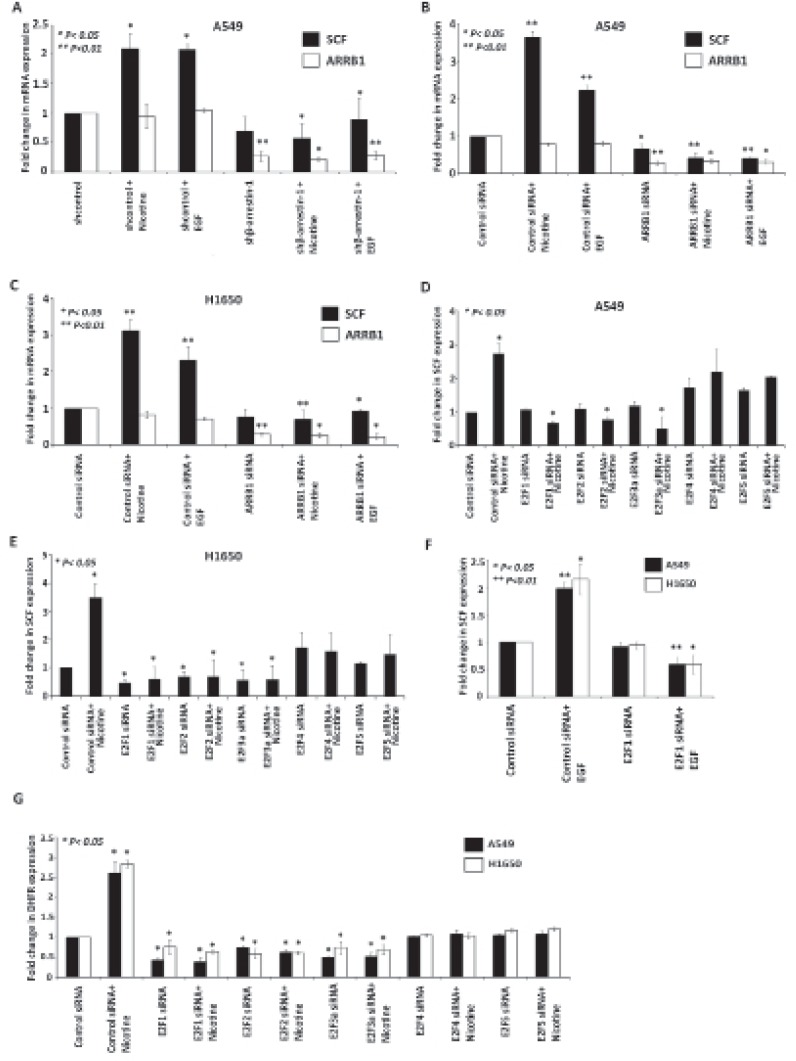
ARRB1/β-arrestin-1 and E2F1 to 3 are necessary for SCF induction by Nicotine and EGF (A) RT-PCR showing the induction of SCF in A549 shcontrol nicotine treated but not in shβ-arrestin1 depleted nicotine treated cells. (B,C) RT-PCR showing the expression of *SCF* in A549 and H1650 NSCLC cells where ARRB1 was knocked down using siRNA prior to nicotine and EGF stimulation. (D, E) RT-PCR showing the effect of siRNA targeting E2F1 to 5 on nicotine-mediated induction of *SCF* in A549 and H1650 cells. (F) RT-PCR showing the effect of siRNA targeting E2F1 on EGF-mediated induction of *SCF*. (G) *DHFR* mRNA levels were significantly reduced by E2F1 to 5 siRNA in A549 and H1650 cells.

Smoking is a well-known risk factor for lung cancer and SCF is known to be aberrantly over expressed in many cancers [27]. Previously we had found that nicotine stimulation of lung cancer cells induced the expression of E2F-regulated proliferative genes through the α7-nicotinic acetylcholine receptors in a β-arrestin-1 as well as Src dependent manner [15]. To ascertain whether a similar pathway facilitates nicotine-mediated induction of SCF, we generated ARRB1/β-arrestin-1 knockout A549 cell line stably expressing β-arrestin-1 specific shRNA (shβ-arrestin-1) and control cells (shcontrol) were generated with A549 cells transfected with empty vector. These stable knockout cells were then treated with nicotine and EGF to see if they can induce SCF. Similarly siRNA to ARRB1/β-arrestin-1 or a non-targeting control siRNA was transfected into A549 and H1650 cell lines. Cells were rendered quiescent by serum starvation for 36 h, followed by 24 h of treatment with nicotine (2 μM) and EGF (100 ng/ml). RT-PCR experiments showed that nicotine and EGF treatment resulted in elevated expression of *SCF* mRNA in control shRNA, control siRNA transfected cells, but not in β-arrestin-1 depleted cells. Depletion of β-arrestin-1 was confirmed by RT-PCR analysis (Figure 2A, 2B and 2C). These experiments suggest that nicotine and EGF induces *SCF* expression in an ARRB1/β-arrestin-1 dependent manner.

### E2F1 transcription factor is necessary for Nicotine and EGF mediated SCF induction

Since our earlier studies had shown that nicotine enhanced E2F1-mediated transcriptional activity [15], attempts were made to assess whether E2F transcription factors are involved in the induction of *SCF*. RT-PCR experiments were conducted on cells transfected with a control siRNA or siRNAs to E2F1 to 5; cells were serum starved for 36 h followed by 24 h of treatment with nicotine (2 μM). As shown in Figure 2D and E depletion of E2F1 to 3 significantly reduced the nicotine-mediated induction of *SCF* in A549 and H1650 cells, but depletion of E2F4 and 5 had no significant effect. Similarly, involvement of only E2F1 was observed for EGF stimulation as well (Figure 2F). Based on this result, further experiments focused only on E2F1 transcription factor and nicotine stimulation. *DHFR* mRNA expression level was used as a control for these experiments (Figure 2G). These results suggest that E2F1 to 3 play a major role in mediating the induction of the *SCF* gene by nicotine and EGF.

**Figure 3:**
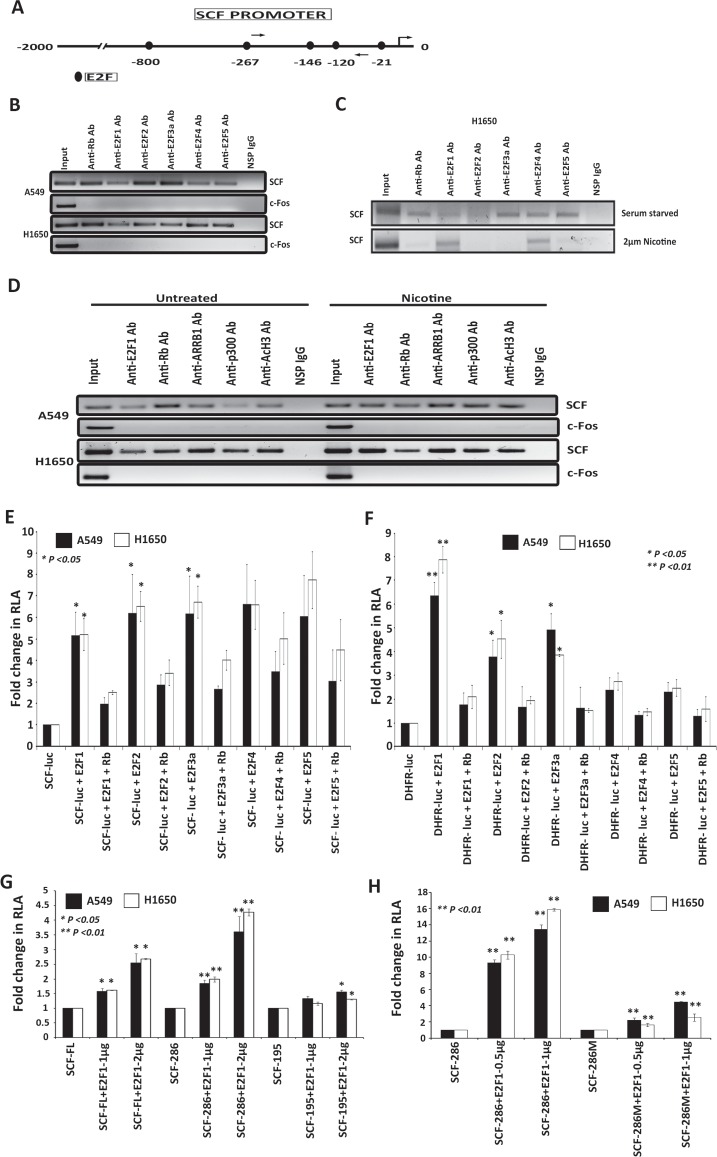
E2Fs regulate the expression of SCF in lung cancer cells (A) Schematic of *SCF* promoter showing putative E2F binding sites; arrows indicate the location of ChIP assay primers. (B) ChIP assays conducted on *SCF* promoter using the E2Fs antibodies. c-Fos promoter was used as a negative control for E2F1 binding and a non-specific IgG was used as a negative control for IP. (C) ChIP assays showing the occupancy of E2F1 and E2F4 on the *SCF* promoter in H1650 cells upon nicotine stimulation. (D) ChIP assays showing the occupancy of E2F1 and ARRB1 on the *SCF* promoter in A549 and H1650 cells upon nicotine stimulation. (E) Transient transfection experiments in A549 and H1650 cells shows that E2Fs could induce SCF promoter and E2F1 to 3 repressed by full length Rb, (F) Transient transfection experiments showing that DHFR is induced by E2F1 to 3 acted as a positive control. (G) Transient transfection results show that SCF-FL and SCF-286bp promoter fragment was E2F1 response while the shortest fragment 195bp that did not have E2F binding sites was not E2F1 responsive. (H) An E2F site mutant of the shortest SCF promoter fragment (SCF286M) showing significantly lower response to E2F1 in co-transfection assays.

Given the above results, we examined the promoter region 2kb upstream of the transcription start site (TSS) of *SCF* gene with the MatInspector (Genomatix) program and this revealed the presence of five E2F binding sites (Figure 3A). Chromatin immunoprecipitation (ChIP) assays were conducted on asynchronously growing A549 and H1650 cells to assess whether E2F1 to 5 and Rb are associated with *SCF* promoter. As shown in Figure 3B, E2F1 to 5 as well as Rb were associated with the *SCF* promoter. There was no Rb or E2F1 to 5 present on the unrelated c-Fos promoter, which was the negative control. There was no amplification in the immunoprecipitation done with an irrelevant antibody, demonstrating the specificity of the assay. This experiment suggests that the E2F sites present on *SCF* promoter can recruit E2Fs and Rb. We performed a ChIP assay in H1650 cell line to assess how the binding of different E2Fs E2F1 to 5, Rb to *SCF* promoter changes upon nicotine stimulation; there were minimal amounts of E2F1 associated with the *SCF* promoter in quiescent cells but stimulation with nicotine enhanced the binding of E2F1 to the promoter, with a reduction in the association of Rb (Figure 3C). There was no involvement of E2F2, 3 and 5 but E2F4 was involved upon nicotine stimulation. ChIP assays were also conducted to assess how the binding of E2F1, Rb and ARRB1/β-arrestin-1 to *SCF* promoter changes upon nicotine stimulation; there were minimal amounts of E2F1 associated with the *SCF* promoter in quiescent cells but stimulation with nicotine enhanced the binding of E2F1 to the promoter, with a reduction in the association of Rb (Figure 3D). Further, as shown in Figure 3D, nicotine stimulation enhanced the association of ARRB1/-arrestin-1 and p300 with the *SCF* promoter in A549 cells, resulting in increased acetylation of Histone H3; these changes were less obvious in H1650 cells. There was no binding observed in lanes immunoprecipitated with the control antibody. These results suggest that nicotine stimulation induces SCF through the mediation of ARRB1/β-arrestin-1 and E2F transcription factors.

Additional experiments were conducted to verify these results. A549 and H1650 cells were transiently transfected with a pGL3-SCF luciferase reporter construct. It was found that cotransfection of E2F1 to 5 led to a significant induction of the *SCF* promoter (Figure 3E); furthermore, cotransfection of the large pocket region of Rb or the full-length Rb repressed the induction mediated by E2F1 to 3. In contrast to the *SCF* promoter, only E2F1 to 3 could induce the proliferative *DHFR* promoter in co-transfection experiments (Figure 3F). We generated shorter promoter fragments of SCF (SCF-286 and SCF-195) and also an E2F1 site mutant of SCF promoter region (SCF286M) in both A549 and H1650 cells. Transient transfection results show that SCF 286bp promoter fragment was E2F1 response while the shortest fragment 195bp that did not have E2F binding sites was not E2F1 responsive (Figure 3G). An E2F site mutant of the shortest SCF promoter fragment (SCF286M) showed significantly lower response to E2F1 in co-transfection assays (Figure 3H). Combined with the siRNA results, we conclude that while over expression of E2F1 to 5 can induce the *SCF* promoter, its regulation is mainly through E2F1 to 3, and we focused on E2F1.

### Contribution of Src, PI3K, MEK and EGFR signaling in regulating SCF expression

**Figure 4:**
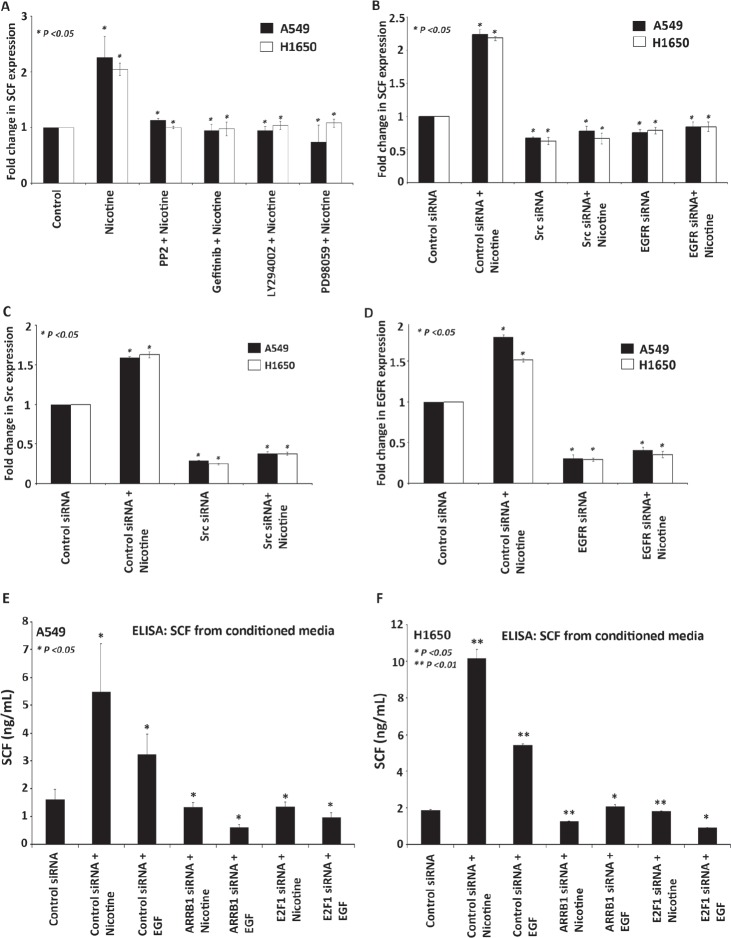
Differential contributions of Src, EGFR, PI3K and MEK signaling in regulating SCF expression (A) Real time PCR showing the inhibition of nicotine-mediated induction of *SCF* by inhibitors of Src (PP2), EGFR (Gefitinib), PI3 kinase (LY294002) and MEK (PD98509). (B-D) siRNAs to Src and EGFR prevented nicotine-mediated induction of *SCF*, as seen by RT-PCR. (E-F) ELISA assay shows increased secretion of SCF in the medium of A549 and H1650 cells treated with nicotine and EGF; levels were significantly reduced in conditioned media from cells transfected with siRNAs to β-arrestin-1 or E2F1.

Experiments were conducted to understand the signaling events that mediate the induction of *SCF* in response to nicotine. We focused on Src, PI3K, MEK and EGFR pathways, since they are known to function downstream of nicotine in different systems. As shown in Figure 4A, stimulation with nicotine induced *SCF* expression in A549 and H1650 cells, as seen by RT-PCR; the stimulation was abrogated in the presence of PP2, LY294002, PD98509 and Gefitinib. The results with inhibitors were confirmed by using siRNAs to Src and EGFR; cells transfected with Src and EGFR siRNA were rendered quiescent for 36 h followed by 24 h of treatment with nicotine (2 μM). As shown in Figure 4B, nicotine mediated induction of *SCF* transcription was significantly decreased in Src as well as EGFR depleted cells. Depletion of *Src* and *EGFR* was confirmed by RT-PCR analysis (Figure 4C and D). Previous studies have already shown that the consequence of unregulated c-Kit activity is subsequently over-activation of downstream signaling pathways like *Src, PI3K, MEK and JAK/STAT* [28]. These studies along with our results show that SCF can respond to various signaling pathways induced by different downstream molecules.

### SCF secretion is induced by nicotine, as seen by ELISA

**Figure 5:**
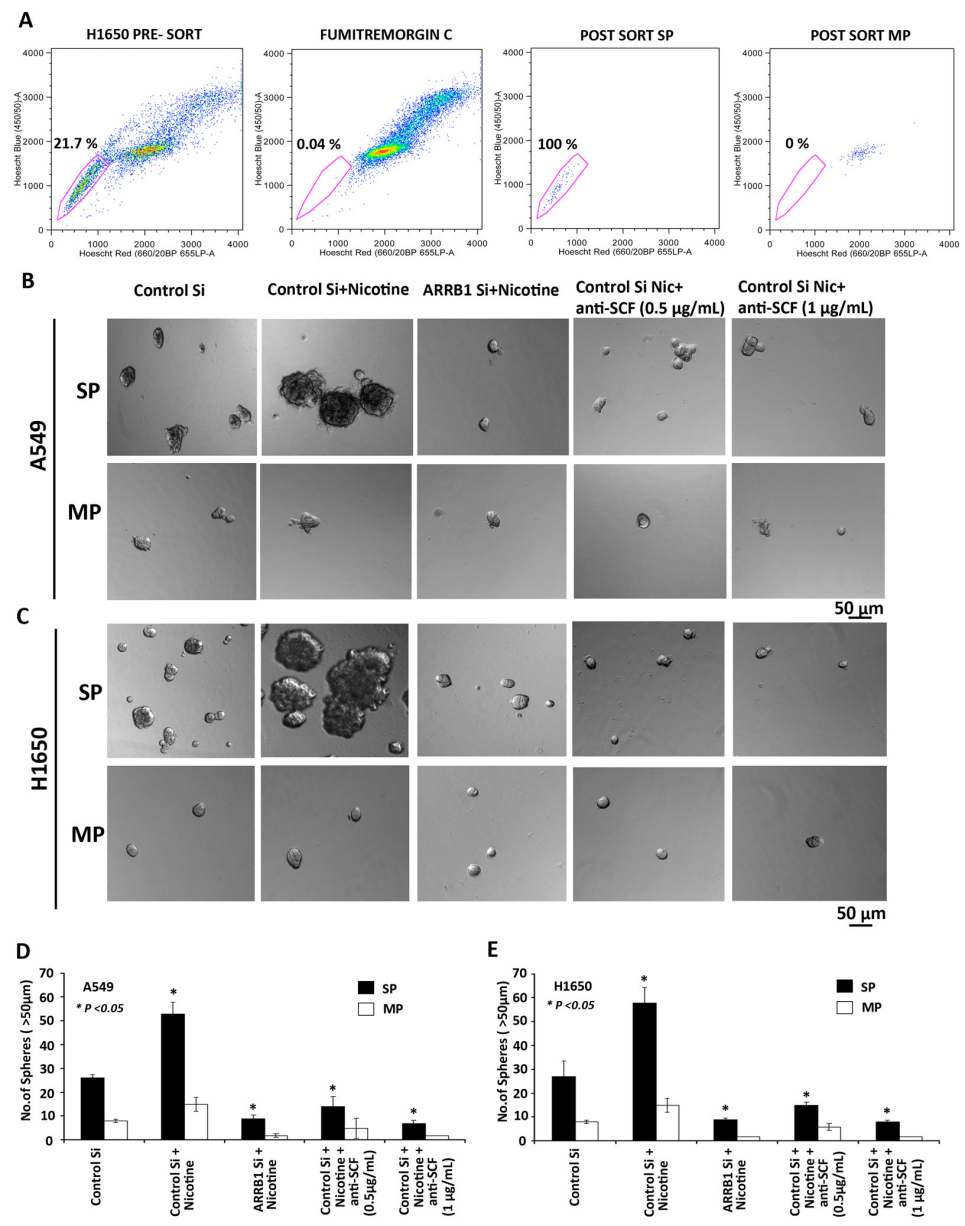
Role of SCF in self-renewal growth of cancer stem-like side population cells (A) Sorting of SP and MP cells in H1650 cells by FACS analysis with Hoechst 33342 dye staining. (B, C) SCF regulates the self-renewal growth of side population cells (SP) and not main population cells (MP) from A549 and H1650 cells. Cell culture supernatants from A549 and H1650 cells transfected with control siRNA, ARRB1/β-arrestin-1 siRNA and stimulated with nicotine was used for sphere formation assay. There was a significant decrease in spheres after treatment with 0.5 and 1μg/ml SCF-neutralizing antibody. (D, E) Total number of spheres generated per well from 2000 cells is plotted.

**Figure 6:**
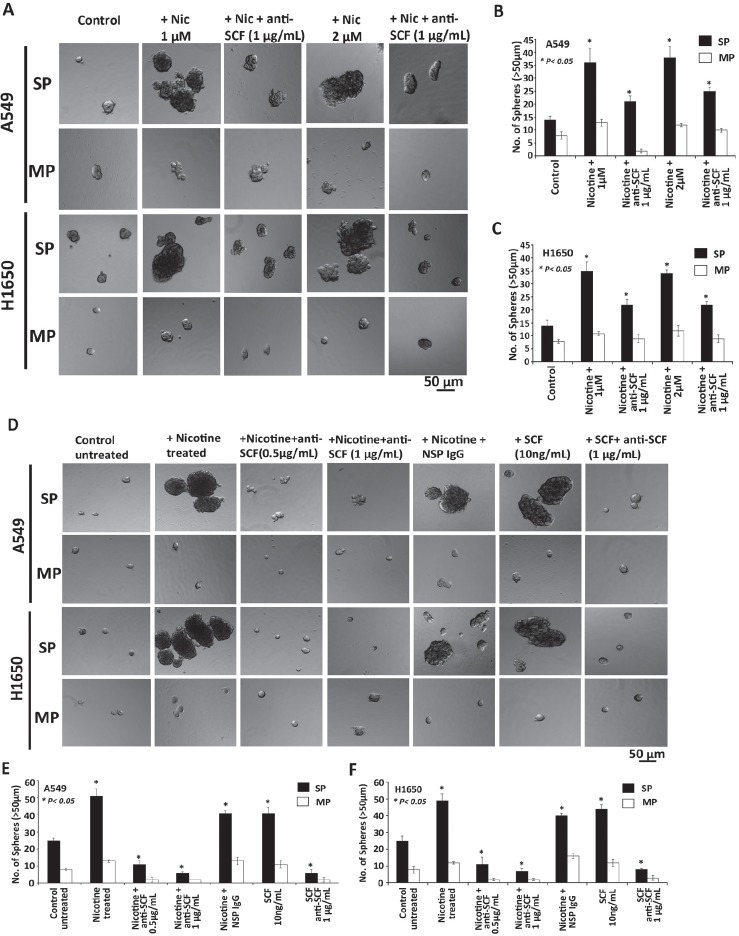
Role of SCF and nicotine in self-renewal growth of cancer stem like side population cells (A) Sphere formation assay in SP and MP cells showing that nicotine independent of SCF can also regulate the self-renewal growth of SP cells but not as significant that of SCF that was confirmed by the addition of SCF neutralizing antibody that resulted in the marginal reduction in the number of spheres in SP cells in both A549 and H1650 cells. (B,C) Total number of spheres generated per well from 2000 cells is plotted. (D) Cell culture supernatants containing the secreted SCF with nicotine treated and untreated in A549 and H1650 cells were used for sphere formation assay in SP and MP cells. Human recombinant SCF (10ng/ml) was used to confirm the role of SCF on stem like SP cells. Results show that SP cells with nicotine induced SCF showed increased formation of tumor spheres and neutralization of SCF with the antibody completely abrogated the growth of spheres in SP. (E, F) Total number of spheres generated per well from 2000 cells is plotted.

It was examined whether reduction in *SCF* message was reflected in the levels of secreted SCF. An ELISA of the conditioned medium from A549 and H1650 cells treated with nicotine and EGF showed increased concentrations of SCF after treatment. Consistent with the above results, the expression of SCF decreased in the conditioned medium from the cells transfected with siRNAs to β-arrestin-1 or E2F1 prior to stimulation with nicotine and EGF, compared to control siRNA transfected cells (Figure 4E and F). The supernatants from the ELISA experiments were used for the sphere formation (self-renewal) assays described below, which were conducted on the SP, MP cells from A549 and H1650 cell lines (Figure 5 and 6).

### SCF regulates the self-renewal of cancer stem-like side population cells in NSCLC

Our earlier studies had shown that ARRB1/β-arrestin-1 can regulate the side population (SP) frequency and self-renewal of stem-like side population cells in NSCL [29] period. Given the finding that nicotine could induce SCF secretion in an ARRB1/β-arrestin-1 dependent fashion and since SCF is known to promote stemness, we examined whether supernatants from nicotine-stimulated cells could promote the self-renewal properties of SP cells. The self-renewal property was examined by performing sphere formation assays using SP and main population (MP) cells isolated from A549 and H1650 cell lines. A representative FACS sorting for H1650 cells is shown in Figure 5A; inclusion of Fumitremorgin C eliminates the SP cells and allowed setting the gate for sorting SP cells [29, 30]. In the first set of experiments, A549 and H1650 cells were transfected with control siRNA or ARRB1/β-arrestin-1 siRNA and rendered quiescent for 36 h followed by 24 h of treatment with nicotine (2 μM). Cell culture supernatants containing the secreted SCF (Figure 3E and F) were harvested and used for sphere formation assay.

SP and MP cells from A549 and H1650 were dissociated into a single-cell suspension, plated (2000 cells/100 μl) in ultralow-attachment plates, and cultured in stem cell selective conditions. SCF supernatant from the transfection experiments and neutralizing SCF antibody (0.5 and 1 μg/ml) was added and incubated for 10 days. SP cells were able to grow as spheres whereas MP cells showed markedly reduced capability suggesting that only SP cells are enriched with cancer stem-like properties. As shown in Figure 5B and C there was increased formation of tumor spheres when SP cells were grown in supernatants from nicotine-stimulated control siRNA transfected cells; inclusion of an anti-SCF antibody prevented the ability of the tissue culture supernatant to induce self-renewal and sphere formation, indicating the involvement of SCF. Interestingly, supernatants from nicotine stimulated ARRB1/β-arrestin-1 siRNA transfected cells did not promote sphere formation in SP cells from either A549 and H1650 cell lines. The average number of spheres (> 50 μm) grown per well is plotted in Figure 5D and E.

Since supernatants from nicotine-stimulated cells promoted self-renewal of SP cells, it was examined whether nicotine could promote self-renewal of SP cells when applied directly. SP cells from A549 and H1650 cells were isolated and treated with 1μM or 2μM of nicotine; it was found that exposure to nicotine could enhance the self-renewal, as seen by sphere-formation assays (Figure 6A). Addition of an anti-SCF antibody significantly reduced the self-renewal of SP cells (Figure 6A to C); this suggests the existence of a feed-forward loop, where nicotine exposure leads to secretion of SCF, which promotes self-renewal of SP cells. In a similar experiment, supernatant from untransfected cells induced self-renewal and could be prevented by an anti-SCF antibody (Figure 6D). As a positive control, recombinant SCF was used to induce sphere formation and this could be inhibited by the anti-SCF antibody in both A549 and H1650 cells (Figure 6E and F). These results suggest that exposure to nicotine induces the expression of SCF, which in turn promotes the self-renewal of stem-like cells in NSCLC, promoting tumorigenesis.

## DISCUSSION

Exposure to tobacco smoke components is strongly associated with the onset of various cancers including those of the lung, pancreas, prostate and the bladder [31-33]. Tobacco specific nitrosamines are known to form DNA adducts, mutating vital genes like K-Ras and p53, facilitating the onset of cancer [34]. Nicotine by itself is not known to have carcinogenic effects in humans or most rodents; at the same time, nicotine has been shown to promote the growth and metastasis of cancers *in vitro* and *in vivo* [35]. Nicotine has been shown to induce epithelial-mesenchymal transition in a variety of cells as well, promoting tumor progression and metastasis [36]. All these effects of nicotine occur through nicotinic acetylcholine receptors, which are expressed on a wide variety of neuronal and non-neuronal cells.

Our earlier studies had shown that nicotine induces cell proliferation through the β-arrestin-1 and Src mediated activation of proliferative pathways, which results in the inactivation of the Rb protein and induction of E2F-mediated proliferative genes. Further, nicotine was found to prevent the pro-apoptotic effects of chemotherapeutic drugs on lung cancer cell lines in a receptor dependent fashion, providing a mechanistic basis for the lower survival rates observed in patients who continue to smoke during chemotherapy [37, 38]. Reports suggest that lung cancer patients who continue to smoke have a shorter median survival compared to nonsmokers and former smokers, particularly when diagnosed with earlier stage (I/II) lung cancer [39]. Since the recurrence of lung cancer in early stage patients leads to poor survival, it is possible that functional effects of nicotine and its receptors on tumor cells could contribute to poorer outcome in smokers.

Cancer stem cells (CSCs) are a subpopulation of undifferentiated cells that are considered to be responsible for tumor initiation, propagation, recurrence and resistance to therapy [40]. They display the ability to self-renew and generate a progeny of the differentiated cells that constitute a large majority of the cells in tumors. Side population cells from lung cancer cell lines have been shown to have cancer stem cell like properties and displayed a specific gene signature [41]; they could form highly metastatic tumors in mouse models. They could self-renew efficiently in sphere-formation assays and showed increased resistance to drugs compared to main population cells [30]. These functions of stem-like side-population cells appear to overlap with the ability of nicotine to promote tumor progression and confer drug resistance. Studies presented in this manuscript provide a mechanistic link by which nicotine can promote stemness and self-renewal of SP cells through the induction of SCF, which can be expected to lead to more aggressive and drug resistant tumors.

SCF is pivotal for the survival of embryonic, fetal and adult stem cells and for their role in generating multiple cell and tissue types during embryonic, fetal and adult life [42]. Stimulation of c-Kit receptor by SCF activates a wide array of signaling pathways and the progression of precancerous stem cells to cancer is associated with an upregulation of c-Kit [43], suggesting that c-Kit-SCF signaling may be involved in the formation and survival of cancer stem cells. SCF also serves as a survival factor to promote cellular proliferation or clonogenic growth in lung cancer [44]. Previous study suggest that SCF–CD117 autocrine signaling could stimulate proliferation of lung CSCs isolated from human NSCLC cell lines in suspension growth [45]. They also show that SCF–c-kit axis is a key regulator for lung cancer stem cell (CSC) survival and proliferation in addition to self-renewal property and provide evidence that blocking stem cell factor (SCF)–c-kit signaling is sufficient to inhibit CSC proliferation and survival promoted by chemotherapy. Interestingly, our results suggest that SCF levels are elevated in adenocarcinomas, but not squamous cell carcinomas; the basis for this difference remains unclear.

**Figure 7:**
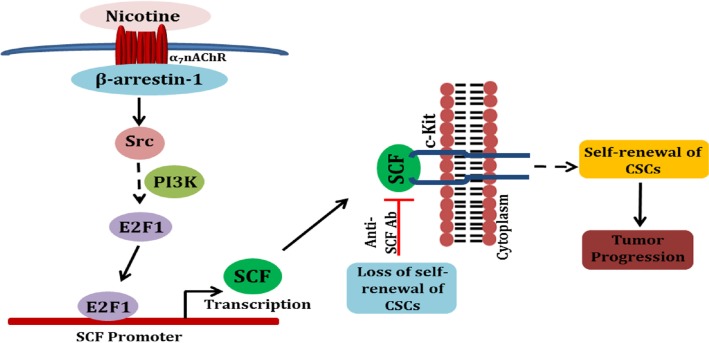
A schematic depicting the potential mechanisms involved in nicotine-mediated enhancement of self-renewal of stem-like side-population cells Binding of nicotine to nAChRs initiates a signaling cascade that involves β-arrestin-1 mediated activation of Src, eventually resulting in E2F1-mediated induction of the SCF promoter. Enhanced secretion of SCF stimulates c-Kit present on stem-like cells, facilitating self-renewal.

It is interesting that the same signaling pathways that are induced by nicotine during cell proliferation contribute to the promotion of self-renewal of stem-like cells. As shown in Figure 7, activation of nAChRs on cancer cells as a result of tobacco smoke exposure leads to the activation of Src in a β-arrestin-1 dependent manner, leading to the activation of E2F1-mediated transcription of the *SCF* gene. The secreted SCF is able to activate the c-Kit receptors on the stem cell population, facilitating their self-renewal. It is also possible that c-Kit, which is expressed on NSCLC cell lines as well as tumors, promotes the growth of the tumors by affecting differentiated, non-stem cells as well. The regulation of SCF expression by Rb and E2F1 raises the possibility that the molecules involved in cell cycle regulation contribute significantly to the stemness as well as progression of tumors. Supporting this hypothesis, an earlier report detected SCF in a microarray experiment among E2F-regulated genes [46]. While these studies on nicotine mediated induction of SCF are relevant to NSCLC in smokers, there are reports suggesting that EGF can also induce SCF in breast cancer cells [47]. Since EGFR mutations are prevalent in NSCLC in non-smokers, it is possible that activation of EGFR might contribute to the induction of SCF in non-smokers as well. At the same time, this particular pathway appears to discriminate the survival profiles of smokers from non-smokers, suggesting a more prevalent role in smokers.

Although only the expression of SCF correlated with smoking other nicotine regulated ARRB1 dependent genes from our microarray data also seem to have important role in various cancers. For instance nicotine induced *MAF* is a B-ZIP transcription factor and *MAF* translocation or overexpression has been observed in human multiple myeloma. Although *cMAF* might function as an oncogene in multiple myeloma, a role for this gene in other cancers has not been shown [48]. Another gene *PTEN* dephosphorylates PIK3CA targets thereby negatively regulating the PI3K–AKT–mTOR pathway, and therefore acting as a potent tumor suppressor [49]. Mutations in *PTEN* have been found in 4–8% of all NSCLC and PTEN mutations are found more commonly in smokers with Squamous cell carcinoma [50]. One of the nicotine suppressed targets *PCDH11Y* is a gene on the human Y chromosome that is known to be selectively expressed in apoptosis and hormone-resistant human prostate cancer cells. It encodes a cytoplasmic protein that interacts with β-catenin and activates WNT signaling pathway [51]. Cancer-testis antigens (CTAs) represent an expanding class of tumor-associated proteins defined on the basis of their tissue-restricted expression to testis or ovary germline cells and frequent ectopic expression in tumor tissue [52]. Recently another member of CTA family synovial sarcoma X chromosome breakpoint (SSX) proteins is considered to be attractive targets for cancer immunotherapy. Although the function of these proteins remains unknown, SSX1/SSX2 overexpression has been known to regulate stem cell migration, suggesting significant role in metastasis in cancer tissue [53].

In conclusion, the studies presented here raise the possibility that exposure to nicotine or its derivatives can promote tumor growth by inducing the expression of SCF and thus modulating the survival and self-renewal of stem-like cells in non-small cell lung cancer, resulting in more progressed and drug resistant tumors often found in smokers. These findings also raise the possibility that targeting pathways that regulate the activity of the E2F1 transcription factor might be effective in combating self-renewal of stem-like cells, thus preventing the growth and progression of tumors.

## MATERIALS AND METHODS

### Cell culture and reagents

A549, H1650 lung adenocarcinoma cancer cell lines were obtained from ATCC and were cultured in F-12K and RPMI (Gibco, Life technologies, USA) containing 10% FBS (Atlanta Biologicals, USA) and 100 U/mL penicillin, and 100 μg/mL streptomycin (from GIBCO). shRNA cell line was made by stably transfecting A549 cells with shRNA construct that specifically targeted β arrestin-1 obtained from an shRNAmir library from Open Biosystems, Huntsville, AL. The studies involving signal transduction inhibitors were done on cells that were rendered quiescent by serum starvation for 36 h, following which cells were treated with the inhibitors for 30 min. Thereafter, cells were stimulated with 2 μM nicotine (Sigma Chemical Company, USA) or 100ng/ml EGF in the presence or absence of the inhibitors for 24 h. The concentrations of inhibitors used for the various experiments were 1 μM PP2, 20 μM PD98059, 500 nM Gefitinib, 10 μM LY294002.

### Microarray analysis

Microarray analysis was performed on A549 cells containing or lacking β-arrestin-1 that were rendered quiescent and subsequently stimulated with nicotine. Total RNA was used to generate cDNA targets, which were hybridized to Human Genome U133A Plus 2.0 oligonucleotide probe arrays (Affymetrix, Santa Clara, CA) according to standard protocols. Raw data was processed by log2 transformation of the expression values, and the mean center expression level for each gene was determined. The microarray data has been deposited in NCBI's Gene Expression Omnibus (GEO) with the accession number GSE44617.

### Analysis of publicly available clinical datasets

Gene expressions profiles analyzed in this study include 22,283 probes quantified with Affymetrix HG-U133A on 360 lung adenocarcinoma samples excluding DFCI samples from Shedden et al [25] and 75 squamous carcinoma samples from Korean SKKU dataset [26]. Raw signal intensities for each probe set as they are contained in the *CEL* files were analyzed using the software package Bioconductor (http://bioconductor.org). Expression values were normalized using MAS5.0 in R. Using mRNA expression profiles of the identified genes as predictors, a prognostic model was constructed using median expression values to stratify patients into low-risk and high-risk groups.

### Statistical analysis

Statistical analyses were done using R package (http://www.r-project.org/). We performed Kaplan-Meier and log-rank test (for *p* value) analysis of overall and recurrence free survival. Overall survival time was calculated from the date of surgery until death or the last follow-up contact. Recurrence free survival time was defined as the time interval between the date of surgery and the date of disease recurrence or death from any cause, whichever came first, or date of last follow-up evaluation. To assess the statistical significance of differences, ANOVA with post hoc student's t test was performed. A *p* value of less than 0.05 was considered to indicate statistical significance, and all tests were two-tailed.

### Immunohistochemistry

A human lung cancer tissue microarray slide (Catalog number IMH-358; Imgenex, San Diego, CA) was immunostained for SCF. The slide contained 59 tissue sections (cores) that included nine normal lung tissues adjacent to the tumors, 40 primary tumors of the lung, and 10 metastatic carcinomas. Immunohistochemical staining for SCF (1:750 dilution, Abcam ab52603) from lung tissues obtained from an orthotopic lung experiment in shcontrol and shβ-arrestin-1 depleted cells treated with nicotine was performed. The mice were administered PBS or nicotine for 6 weeks. The immunohistochemical staining was performed as described [15] and the samples were scored by a pathologist (D. Coppola). The semi quantitative score was reached by taking into consideration both cellularity and intensity of expression (semi quantitative score = cellularity × intensity). Cellularity was scored as follows: a score of 3 equals to greater than 66% cellularity, a score of 2 equals to 34%–65% cellularity, and a score of 1 equals to less than 33% cellularity. Intensity was scored as follows: a score of 3 equals to strong intensity, a score of 2 equals to moderate intensity, and a score of 1 equals to weak intensity. The score of 1 or above was considered as positive expression of SCF. The images were captured at 20X and 40X magnification.

### siRNA transfections and real-time PCR

siRNA for β-arrestin-1, E2F1, c-Src and EGFR were purchased from Santa Cruz Biotechnology Inc. A non-targeting siRNA sequence was used as control. 100 pmol of siRNAs (Santa Cruz Biotechnology, USA) were transfected using Oligofectamine. For real-time PCR (RT-PCR), total RNA was isolated by RNeasy miniprep kit (QIAGEN, USA) according to the manufacturer's protocol. Real-time PCR was done with 1 μL of the reverse transcription product in a MyiQ real-time PCR detection system (Bio-Rad) by using iQ SYBR Green PCR Supermix (Bio-Rad, USA). Data were analyzed by ΔΔCt method, where the gene of interest was normalized to GAPDH and then compared with the non-targeting siRNA control sample.

Primers for SCF were synthesized by IDT DNA Technologies. A 117 bp fragment of SCF at its 3′ end was amplified using the following primers:

Forward: (5′-TGCGCTCGGGCTACCCAATG-3′)
Reverse (5′-AGCGCTGCGATCCAGCACAAA-3′).

The endogenous human glyceraldehyde-3-phosphate dehydrogenase (GAPDH) was used as control and was amplified using the following primers:

Forward: (5′-GGTGGTCTCCTCTGACTTCAACA-3′)
Reverse: (5′-GTTGCTGTAGCCAAATTCGTTGT-3′).

### Generation of SCF promoter mutant constructs

Shorter fragments of SCF promoter was PCR amplified using the following primer pairs to generate promoter constructs containing 286 bp and 195 bp DNA sequence upstream of TSS.

SCF-pro-F(286)-GCGAGCAGTAGCTGCAGGGTACC
SCFproR(171)- GCGCTGCGATCCAGCACAAACAG
SCF-proF(195)- AGCGGACAAGGTTGGCCTAATCTG
SCFproR(171)- GCGCTGCGATCCAGCACAAACAG

The PCR products were subsequently cloned into pGL3 vector. The E2F binding sequence in the SCF promoter (−286) GCGTCCCCGCGCCTTCGCGGTCTCCCGCCAGTG (−249 to −233 & −233 to −216) was mutated to GCGTCCCCATATCTTCGCGGTCTCCATATAGTG by changing the core sequences GCGC and CGCC to ATAT using site directed mutagenesis kit (Clonetech, USA).

### Transient transfections and luciferase assays

Plasmid containing genomic DNA fragments of the human *SCF* gene 5′ -flanking region, spanning from +184 to - 2185 (pGL3-SCF-Luc) relative to the transcription initiation sites was a kind gift from Dr. Zhong Chao Han, Tianjin, China. A549 and H1650 cells were transfected with 0.5 μg of SCF reporter along with 1 μg E2F1 to 5 and 2 μg full-length Rb using FuGENE HD (Roche, USA). Cotransfection with 0.5 μg of pRL construct containing Renilla reniformis luciferase gene was used for normalization and luciferase assays were conducted by the Dual Luciferase Assay System (Promega, USA). Similarly transfections assays were performed with the SCF promoter mutant constructs. Relative luciferase activity was defined as the ratio of firefly luciferase activity to Renilla luciferase activity.

### Chromatin immunoprecipitation (ChIP) analysis

Chromatin immunoprecipitation (ChIP) assays were conducted on A549 and H1650 cell lines, using 2.5 × 10^7^ cells per immunoprecipitation (IP) reaction, as per our published protocols [15]. The following primary antibodies were used at 5 μg concentration for each ChIP reaction: rabbit anti-human E2F1, mouse anti-human Rb1, rabbit anti-human EP300, mouse anti-human-ARRB1 (Santa Cruz Biotechnology) and rabbit anti-human acetylated histone H3 (Upstate Biotechnology, USA). Rabbit anti-mouse IgG antibody was used as control. The PCR primers used are as follows: E2F1 (−264 from TSS) forward primer, 5′- GCGCGAGGTATTTCGTCTGT -3′; E2F1 (−56 from TSS) reverse primer, 5′ - ATGCCCCAGAAGTTTGGCAG -3′. PCR for c-Fos was used as the control in all experiments. The E2F binding sequences within the SCF promoter are as follows: GTTGGTCAAGAAAATTAA (−800 from TSS), GCGCGAGGTATTTCGT (−267 from TSS), GCGGTCTCCCGCCAGTG (−120 from TSS) and TCCCGATTCTTCCCTCC (−21 from TSS).

### ELISA for SCF

A549 and H1650 cells transfected with the β-arrestin-1 (ARRB1) and E2F1 siRNAs were rendered quiescent for 36 h followed by 24 h of treatment with nicotine (2 μM). Cell culture supernatants containing the secreted SCF were concentrated using Pierce protein concentrators, 9K MWCO (Thermo Scientific, USA). ELISA for SCF was performed according to the manufacturer's instructions (Human KITLG, KA1022, Abnova, Taiwan). Biotinylated secondary antibody and streptavidin conjugated horseradish peroxidase were used for detection of captured SCF by measuring absorbance at 450 nm, using a 96-well plate spectrophotometer (BioTek, VT, USA).

### Hoechst 33342 dye efflux assay for side-population cell analysis and cell sorting

Stem-like side population cells were isolated from A549 and H1650 cells by FACS using Hoechst 33342 dye exclusion as described by Goodell et al [54] with modifications [29, 30] Hoechst 33342 dye was excited at 350 nm and its fluorescence was analyzed using 400-500 nm BP filter for blue emission and 640-680 nm BP filters in combination with 655 nm LP-filter for red emission. Data were acquired using FACS Vantage (DiVa), and sorted using FACS Vantage (DiVa) cell sorter and analyzed using FlowJo software (Tree Star).

### Sphere formation (self-renewal) assays

Sorted SP and MP cells from A549 and H1650 were plated in 96 well plates at the density of 2000 cells/well in total 200 μl medium [30]. 2000 cells in 100 μl serum free stem cell selective media (DMEM/F12K (1:1) (Invitrogen), supplemented with 1X N2 supplement (Invitrogen) and remaining 100 μl containing secreted SCF supernatant from the transfection experiments were added and allowed to grow as spheres for 10 days. Images of the spheres were taken using phase contrast microscope (Nikon) and total numbers were counted.
